# Novel dual‐action prodrug triggers apoptosis in glioblastoma cells by releasing a glutathione quencher and lysine‐specific histone demethylase 1A inhibitor

**DOI:** 10.1111/jnc.14655

**Published:** 2019-02-03

**Authors:** Martin Engel, Yi Sing Gee, Dale Cross, Alan Maccarone, Benjamin Heng, Amy Hulme, Grady Smith, Gilles J. Guillemin, Brett W. Stringer, Christopher J. T. Hyland, Lezanne Ooi

**Affiliations:** ^1^ Illawarra Health and Medical Research Institute Wollongong New South Wales Australia; ^2^ School of Chemistry and Molecular Bioscience University of Wollongong Wollongong New South Wales Australia; ^3^ Mass Spectrometry User Resource and Research Facility School of Chemistry University of Wollongong Wollongong New South Wales Australia; ^4^ Faculty of Medicine and Health Sciences Macquarie University Sydney New South Wales Australia; ^5^ QIMR Berghofer Medical Research Institute Herston Queensland Australia

**Keywords:** apoptosis, glioblastoma, LSD1, methylation, oxidative stress

## Abstract

Targeting epigenetic mechanisms has shown promise against several cancers but has so far been unsuccessful against glioblastoma (GBM). Altered histone 3 lysine 4 methylation and increased lysine‐specific histone demethylase 1A (LSD1) expression in GBM tumours nonetheless suggest that epigenetic mechanisms are involved in GBM. We engineered a dual‐action prodrug, which is activated by the high hydrogen peroxide levels associated with GBM cells. This quinone methide phenylaminecyclopropane prodrug releases the LSD1 inhibitor 2‐phenylcyclopropylamine with the glutathione scavenger *para*‐quinone methide to trigger apoptosis in GBM cells. Quinone methide phenylaminocyclopropane impaired GBM cell behaviours in two‐dimensional and three‐dimensional assays, and triggered cell apoptosis in several primary and immortal GBM cell cultures. These results support our double‐hit hypothesis of potentially targeting LSD1 and quenching glutathione, in order to impair and kill GBM cells but not healthy astrocytes. Our data suggest this strategy is effective at selectively targeting GBM and potentially other types of cancers.

**Open science badges:**

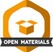

This article has received a badge for *Open Materials* because it provided all relevant information to reproduce the study in the manuscript. The complete Open Science Disclosure form for this article can be found at the end of the article. More information about the Open Practices badges can be found at https://cos.io/our-services/open-science-badges/.

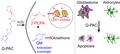

**Abbreviations used**
2‐PCPA: *trans*‐2‐phenylcyclopropylamine
4‐OHT: 4‐hydroxytamoxifen
CPA: cyclopropylamine
GBM: glioblastoma
GSH: glutathione
H3K4: histone 3 lysine 4
LSD1: lysine‐specific histone demethylase 1A
NMR: nuclear magnetic resonance
QAC: quinone methide aminocyclopropane
QM: quinone methide
Q‐PAC: quinone methide phenylaminocyclopropane
ROS: reactive oxygen species

Glioblastoma (GBM) is the most common primary and malignant brain tumour in adults. GBM is an aggressive tumour that proliferates and migrates rapidly (Demuth and Berens 2004), leaving patients with a median survival of 12–15 months (McLendon and Halperin 2003; Verhaak *et al*. 2010). To improve treatment outcomes, the search for new targets against GBM has turned towards the contribution of epigenetic mechanisms to GBM formation and progression (Mack *et al*. 2015). Transcriptional activation and repression of tumorigenesis‐relevant genes is influenced by local histone methylation. For example, methylation at histone 3 lysine 4 (H3K4) residues, which is associated with increased gene expression (Santos‐Rosa *et al*. 2002; Liang *et al*. 2004; Schneider *et al*. 2004), is reduced in severe GBM cases (Liu *et al*. 2010). The catalysing enzymes of these non‐permanent epigenetic markers are thus of particular interest for therapeutic approaches.

Lysine‐specific histone demethylase 1A (LSD1) belongs to the flavin adenine dinucleotide‐dependent amine oxidase family and catalyses the demethylation of monomethylated (me1) and dimethylated (me2) H3K4 residues (Shi *et al*. 2004; Forneris *et al*. 2005). In combination with transcription factors, such as repressor element 1‐silencing transcription factor, LSD1 activity and the removal of H3K4 methylation are associated with gene repression (Ooi and Wood 2007). In concert with reduction in H3K4 methylation, GBM cells have increased LSD1 protein levels (Singh *et al*. 2011; Sareddy *et al*. 2013; Zheng *et al*. 2015), with evidence for the functional involvement of LSD1 in GBM proliferation (Suvà *et al*. 2014). Positive results from pharmacological inhibition of LSD1 in other tumours with increased LSD1 expression, such as leukaemia, lung and breast cancers (Fiskus *et al*. 2014; Kumarasinghe and Woster 2014; Murray‐Stewart *et al*. 2014; Mohammad *et al*. 2015), indicate a promising treatment potential for GBM by targeting LSD1 inhibition.

To increase the prospective success of a LSD1 inhibitor‐based GBM drug, employment of a cancer‐selective mechanism is needed to reduce undesired effects in healthy tissue. Cancer cells produce higher levels of reactive oxygen species (ROS), including H_2_O_2_, than non‐cancerous cells (Reuter *et al*. 2010), while being able to withstand 10‐ to 100‐fold higher H_2_O_2_ concentrations (Hagen *et al*. 2012). Activation of a prodrug by high H_2_O_2_ levels can therefore reduce off‐target effects in healthy tissue (Peng and Gandhi 2012; Singer *et al*. 2015). Hagen *et al*. (2012) have elegantly shown the activation of an aminoferrocene‐based anti‐cancer prodrug by H_2_O_2_, which takes advantage of high extracellular H_2_O_2_ levels in close proximity to cancer cells to increase selectivity (Zieba *et al*. 2000; Lim *et al*. 2005). Their prodrug undergoes oxidative break down to release glutathione (GSH)‐scavenging quinone methide (QM) and a Fe catalyst for ROS generation, which work in concert to amplify accumulation of ROS in cancer cells (Hagen *et al*. 2012). Similarly, Noh *et al*. (2015) developed a dual stimuli‐responsive anti‐cancer prodrug activated by H_2_O_2_ and acidic conditions to generate QM and ROS‐generating cinnamaldehyde. Importantly, cancer cells survive high ROS by up‐regulation of the ROS scavenger GSH (Ogunrinu and Sontheimer 2010). Both these prodrugs and other anti‐cancer drugs therefore directly or indirectly target the GSH mechanism to further elevate intracellular ROS and kill their target cells (Alexandre *et al*. 2006; Badr *et al*. 2013; Kohsaka *et al*. 2013; Noh *et al*. 2015). However, cancer‐cell‐selective prodrugs based on the common LSD1 inhibitor *trans*‐2‐phenylcyclopropylamine (2‐PCPA/tranylcypromine) are almost unexplored to date. The group of Suzuki made a seminal contribution in this field, with their development of a prodrug that conjugated 2‐PCPA with the anti‐oestrogen agent 4‐hydroxytamoxifen (Ota *et al*. 2016). Their prodrug selectively released 4‐hydroxytamoxifen in the presence of LSD1, which has increased expression in breast cancer tissue (Lim *et al*. 2010). As such, the prodrug was able to inhibit the growth of breast cancer cells via inhibition of LSD1 and oestrogen receptor alpha, while not exhibiting cytotoxicity towards normal cells.

Advancing on existing (pro)drugs that focus primarily on amplification of oxidative stress, we have developed a dual‐action prodrug that releases the LSD1 inhibitor 2‐PCPA and the GSH scavenger QM following H_2_O_2_ activation via an aryl boronate trigger. This dual‐action prodrug was more effective than either 2‐PCPA or QM precursors alone or when applied together as independent compounds. The quinone methide phenylaminocyclopropane prodrug (Q‐PAC) impaired key GBM cell behaviours and triggered cell apoptosis through its hybrid action in several primary and immortal GBM cell cultures. Our data support the double‐hit hypothesis of targeting LSD1 and scavenging GSH, in order to selectively impair and ultimately kill GBM cells over healthy astrocytes.

## Materials and methods

### Chemical synthesis and analysis

All reactions were conducted in oven‐dried glassware under nitrogen atmosphere. Reaction solvents were dried by passing through a column of activated alumina and then stored over 4 Å molecular sieves. Progress of reactions was tracked by thin‐layer chromatography (TLC) and was performed on aluminium‐backed silica gel sheets (Grace Davison, Columbia, MD, USA, UV254). TLC plates were visualized under UV lamp at 254 nm and/or by treatment with one of the following TLC stains: phosphomolybdic acid stain: phosphomolybdic acid (10 g), absolute EtOH (100 mL); Potassium permanganate stain: KMnO_4_ (1.5 g), 10% NaOH (1.25 mL), water (200 mL); Vanillin stain: Vanillin (15 g), concentrated H_2_SO_4_ (2.5 mL), EtOH (250 mL). Column chromatography was performed using silica gel (40–75 μm) as the solid phase. For nuclear magnetic resonance (NMR) spectroscopy, analytes were dissolved in deuterated chloroform unless stated otherwise. NMR spectra for each compound were collected from one of the following instruments: Mercury 2000 spectrometer operating at 500 and 125 MHz for ^1^H and ^13^C NMR, respectively, or a Varian spectrometer operating at 300 and 75 MHz for ^1^H and ^13^C NMR, respectively. NMR data are expressed in parts per million (ppm) and referenced to the residual chloroform in the deuterated solvent (7.26 ppm for ^1^H NMR and 77.16 ppm for ^13^C NMR). The following abbreviations are used to assign the multiplicity of the ^1^H NMR signal: s = singlet; bs = broad singlet; d = doublet; t = triplet; q = quartet; quin = quintet; dd = doublet of doublets; m = multiplet. For mass spectrometry analytes were dissolved in HPLC grade methanol. High‐resolution mass spectra were collected from a Waters Xevo G1 QTOF mass spectrometer [Rydalmere, NSW, Australia, electrospray‐ionization mass spectrometry (ESI‐MS) or atmospheric solids analysis probe mass spectrometry (ASAP‐MS)] or Thermo Fisher Scientific Australia (North Ryde, NSW, Australia) LTQ Orbitrap XL (ESI). Infrared spectra were obtained from a Shimadzu IRAffinity‐1 Fourier transform infrared spectrophotometer with an ATR attachment (Shimadzu, Kyoto, Japan). The log BB value for Q‐PAC was calculated using ChemDraw Professional 15.0 (PerkinElmer, Hopkinton, MA, USA).

#### Synthesis details of Q‐PAC (4‐(4,4,5,5‐tetramethyl‐1,3,2‐dioxaborolan‐2‐yl)benzyl (2‐phenylcyclopropyl)carbamate)

Triethylamine (0.4 mL, 290.4 mg, 2.87 mmol, 1.2 equiv) and diphenylphosphoryl azide (0.58 mL, 742.4 mg, 2.70 mmol, 1.1 equiv) were added to a solution of 2‐phenylcyclopropane‐1‐carboxylic acid (399.6 mg, 2.46 mmol, 1 equiv) and 4‐(hydroxymethyl)phenylboronic acid pinacol ester (637.6 mg, 2.72 mmol, 1.1 equiv) in dry dioxane (5 mL). The reaction solution was heated at 105°C for 4 h then cooled to 22°C. Solvent was evaporated under reduced pressure and the compound was purified by column chromatography (25% ethyl acetate in hexane). The title compound was obtained as a colourless oil (513.6 mg, 1.31 mmol) in 53% yield.

^1^H NMR (500 MHz, CDCl_3_): δ 7.79 (d, *J *=* *7.5 Hz, 2H), 7.33 (d, *J *=* *7.5 Hz, 2H), 7.25–7.23 (m, 2H), 7.17–7.09 (m, 3H), 5.23 (bs, 1H), 5.12 (s, 2H), 2.75 (bs, 1H), 2.06 (bs, 1H), 1.33 (s, 12H) and 1.18 (bs, 2H) ppm. ^13^C NMR (75 MHz, CDCl_3_)^a^: δ 156.8, 140.5, 139.5, 135.1, 128.4, 127.2, 126.6, 126.2, 83.9, 66.7, 32.7, 24.9^b^ and 16.2 ppm. IR (Neat): 3318, 2977 and 1706 cm^−1^. High resolution mass spectrometry (HRMS) (ESI) Found: M+, 393.2102. C_23_H_28_BNO_4_ requires M+, 393.2111.

Synthesis of quinone methide aminocyclopropane (QAC) is included in the supplemental methods. 4‐(hydroxymethyl) PhenylBoronic acid pinacol Ester was purchased from AKScientific (AMTB135; Union City, CA, USA), 2‐PCPA from Sigma‐Aldrich (P8511; Castle Hill, NSW, Australia).

### Prodrug activation assay

Following the procedure of Hagen *et al*. (2012) a solvent system of 9 : 1 acetonitrile : water (v : v) was used to prepare a solution of Q‐PAC and triethylamine (both 0.9 mM) for activation with hydrogen peroxide (9 mM). At 5 min intervals out to 30 min aliquots were diluted 90‐fold in solvent for analysis via electrospray ionization mass spectrometry on either a Thermo LTQ ion‐trap or LTQ Orbitrap XL. Both utilized an Ion Max ESI source operated in positive mode with nitrogen as the desolvation gas. The following conditions were employed on the single‐trap instrument: 5 μL/min infusion rate, 3.5 kV source voltage; sheath, auxiliary and sweep gases set to 12, 0 and 0 (arbitrary flow), respectively; capillary temperature 200°C and voltage 46 V; tube lens 130 volts. Settings for Orbitrap analysis: 10 μL/min infusion rate, 4.2 kV source voltage; sheath, auxiliary and sweep gases set to 10, 0 and 0 (arbitrary flow), respectively; capillary held at 275°C and 50 volts; tube lens 150 volts. The infusion syringe, tubing and ESI probe were rinsed with solvent until the ionized Q‐PAC signal was reduced to background levels prior to analysis of a particular sample. Spectra reported here constitute the average between 50 and 100 scans and were analysed to monitor reaction species relative to Q‐PAC as a function of reaction time.

### LSD1 inhibition assay

The effect of Q‐PAC on the demethylase activity of LSD1 was assessed *in vitro* using the fluorometric LSD1 Assay Kit (# 700120; Cayman Chemical, Ann Arbor, MI, USA) according to the manufacturer's instructions. Each concentration was assessed in duplicate alongside no inhibitor controls and enzyme‐only reactions, with the average fluorescence intensity from three consecutive measurements used (FLUOstar Optima; BMG Labtech (Mornington, Vic., Australia), excitation/emission 540 nm/580 nm).

### Cell culture assays

#### Culture details

*U87MG* cells (ECACC Cat# 89081402, RRID:CVCL_0022, Acc Nr.: 89081402, obtained in 2014, Female astrocytoma, identity confirmed via short tandem repeat profiling by Garvan Institute (Sydney, NSW, Australia) in 2015) were maintained in Dulbecco's Modified Eagle Medium with F12 supplement (Life Technologies, Carlsbad, CA, USA, #10565‐018), 10% foetal bovine serum (Bovogen, Keilor East, Vic., Australia, #SFBS‐F) and seeded at 20 000 cells/cm^2^. Cells were used between passages 8 and 15, absence of mycoplasma confirmed every 3 months (MycoAlert; Lonza, Basel, Switzerland). The U87MG line is listed by ICLAC for contamination of the ATCC version. We used the ECACC version in this study, which shows to be not identical to the ATCC version, or was contaminated with other cell lines based on our short tandem repeat profiling analysis.

*Primary glioblastoma cultures* provided by the Brain Cancer Research Unit of the QIMR Berghofer Medical Research Institute (2015) were established from untreated biopsy samples of different glioblastoma subtypes (Verhaak *et al*. 2010; Day *et al*. 2013) (SJH1: 72 years male, neural; RN1: 56 years male, classical; JK2: 75 years male, proneural). Approval for this study was obtained from the Human Research Ethics Committee of The University of Wollongong (HE16/324). Cells were maintained in Knockout‐Dulbecco's modified Eagle's medium/F12 (Life Technologies, #12660‐012) with StemPro supplement (Life Technologies, #A10508‐01), human epidermal growth factor (20 ng/mL) (Life Technologies, #PHG0314) and human FGF2 (10 ng/mL) (Life Technologies, #PHG0024), and seeded at 35 000 cells/cm^2^ on matrigel (Corning, NY, USA, #354277, 1/100 dilution). Cells were used between passages 5 and 13 in 2015 and 2016, absence of mycoplasma confirmed every 3 months (MycoAlert, Lonza).

*Human astrocyte cultures* were generated from human foetal brain tissue, which was obtained from 17‐ to 20‐week‐old foetuses collected after therapeutic termination following informed consent. Approval for this study was obtained from the Human Research Ethics Committee of Macquarie University (#5201200411). Written informed consent was obtained from the participants. Astrocytes were prepared using a protocol adapted from previously described methods (Guillemin *et al*. 1997) with slight modification. One gram of brain was washed thrice with phosphate‐buffered saline (PBS) containing 1% antibiotic/anti‐mycotic to remove contaminating blood. Visible blood vessels were removed with sterile scissors. Next, the tissue was placed in RPMI medium (Sigma‐Aldrich) supplemented with 10% fetal bovine serum and 2% antibiotic/anti‐mycotic and dissociated mechanically by pipetting with a serological pipette. After 1 week in culture, the medium was removed and the culture was washed with PBS to remove unattached tissue fragments followed by addition of fresh medium. Once confluent, the culture was subjected to successive passage with trypsin‐EDTA (0.25%) (Life Technologies) to remove contaminating cells and seeded at 20 000 cells/cm^2^ for experiments. Cells were used between passages 2 and 5 in 2016, and absence of mycoplasma was confirmed after collection (MycoAlert, Lonza).

#### Confluence assay

Culture confluence was monitored in 96‐well plates (Greiner Bio‐One, Kremsmünster, Austria) imaged every 2 h using an IncuCyte Zoom (Essen Bioscience, Ann Arbor, MI, USA) at 10× magnification (1.22 μm/pixel resolution) with three images per well. Pre‐ and post‐treatment confluence was quantified through the inbuilt basic analyser algorithm (Essen Bioscience) adjusted to the individual morphology of each culture type.

#### Migration assay

For migration assays, cells were seeded into ImageLock 96‐well Plates (Essen Biosciences) and maintained until 70% confluent. The 700–800 μm scratch wounds were made in each well using the 96 well WoundMaker (Essen Biosciences) directly prior to drug treatment. Plates were imaged every 2 h and migration into the wound area was quantified using the inbuilt Scratch Wound algorithm (Essen Biosciences), adjusted to the individual morphology of each culture type.

#### Invasion assay

Cell invasion was examined in real time using the xCELLigence RTCA DP System (Roche Applied Science, Penzberg, Germany). The xCELLigence system (Roche Applied Science) allows continuous quantitative monitoring of cellular behaviour including invasion by measuring electrical impedance at a porous membrane (pore size 8 μm). U87 cells were seeded at 22 500 cells/well into specialized two‐layer cell invasion and migration plates coated with 20 μL matrigel (Corning, 1 : 30 dilution) and cultured without FBS for 24 h in the presence or absence of Q‐PAC. Lower layer wells were filled with Dulbecco's modified Eagle's medium/F12 with 10% FBS as chemoattractant. Invasion was continuously monitored in real time over a period of 24 h. Data analysis was carried out using RTCA Software 1.2.1 (Roche Applied Science) supplied with the instrument.

#### Apoptosis assay

For apoptosis assays, the culture media were supplemented with caspase 3/7 NucView 488 enzyme substrate (2.5 μM final concentration; Biotium, Fremont, CA, USA, #10402) 2 h prior to drug treatment. Phase‐contrast and fluorescent images were captured using an IncuCyte Zoom (green emission/excitation at 460 nm/524 nm) at 2 h intervals and 10× magnification. Caspase substrates were quantified using the inbuilt basic analyser algorithm (Table 1) from a minimum of three images per well and time point.

**Table 1:** Mask parameters for Incucyte Basic analyser image analysis

Targets	Channel	Exposure (ms)	Background correction	Edge sensitivity	Minimum particle size (μm^2^)	Maximum particle size (μm^2^)
Caspase 3/7 Substrates	Green	400	Top‐Hat (10 μm, 2 GCU)	0	10	∞
MCM2	Green	400	Top‐Hat (20 μm, 0.4 GCU)	0	7	∞, maximum eccentricity: 0.96
Reddot2	Red	800	Top‐Hat (20 μm, 0.3)	−11	15	∞

### Cell viability assay

Culture viability was assessed with the resazurin‐based Presto Blue cell viability reagent (Life Technologies, #A13261) according to the manufacturer's instructions (2 h incubation) and quantified on a FLUOstar Optima (BMG Labtech, excitation/emission 540 nm/580 nm).

### Immunocytochemistry

Cultures were fixed (4% paraformaldehyde, 15 min) and blocked (5% goat serum, 1 h) before incubation with MCM2 polyclonal rabbit antibody [Cell Signaling Technology, Beverly, MA, USA, #4007, RRID:AB_2142134, 1 : 500 in 5% bovine serum albumin (BSA)] overnight at 4°C. This was followed by incubation with goat anti‐rabbit IgG conjugated to Alexa 488 (Life Technologies, #A11008, RRID:AB_143165, 1 : 1000, 1% BSA) for 1 h at 22°C and reddot2 (Biotium, #40061‐1, 1/200) as a nuclear counterstain. Images were captured on an Incucyte Zoom in phase‐contrast, green and red (emission/excitation 585 nm/635 nm) at 20× magnification with three images per well. The fraction of MCM2‐positive cells was determined through automatic counting of reddot2 and MCM2‐positive cells per image (see Table 1 for mask parameters).

### Western blot

For histone modification quantification, cultures were lysed in triton extraction buffer (PBS containing 0.5% Triton X 100 (v/v), 1% protease inhibitor cocktail (P8340‐1ML; Sigma‐Aldrich) and 0.02% (w/v) NaN_3_) and histones extracted in 0.2 M HCl at 4°C over 16 h. Reduced samples were separated on 15% polyacrylamide gels and transferred onto polyvinylidene difluoride membranes (Millipore Corporation, Bedford, MA, USA). Membranes were immunoblotted at 4°C over 16 h for monomethylated H3K4 (5% milk block; Abcam, Cambridge, UK, #ab8895, RRID:AB_306847, 1 : 10 000 in 1% BSA), dimethylated H3K4 (5% milk block; Abcam, #ab7766, RRID:AB_2560996, 1 : 10 000 in 1% BSA) and acetylated H4 (3% milk block; Millipore #06‐866, RRID:AB_310270, 1 : 4000 in 3% milk) followed by goat anti‐rabbit IgG‐horseradish peroxidase (Millipore, #AP307P, RRID:AB_11212848, 1 : 2500 in 1% milk) and detected by chemiluminescence. LSD1 expression was quantified in whole‐cell lysates, separated on Criterion TGX Stain‐Free Precast Gels (4–20%; Bio‐Rad Laboratories, Hercules, CA, USA, #567‐8095) and transferred onto polyvinylidene difluoride membranes. Total protein loading was quantified by UV imaging of trihalo transferred from the gels. Membranes were immunoblotted at 4°C over 16 h for LSD1 (5% milk block; Cell Signaling Technology, #C69G12, RRID:AB_2070132, 1 : 1500 in 5% milk), followed by goat anti‐rabbit IgG‐horseradish peroxidase (Sigma, St Louis, MO, USA, #A0545, RRID:AB_257896, 1 : 3000 in 2.5% milk) and detected by chemiluminescence.

### GSH assay

Reduced GSH of cell lysates was measured with a fluorometric kit, according to the manufacturer's instructions (Abcam, #ab138881) and fluorescence intensity monitored on a FLUOstar Optima (excitation/emission 490 nm/520 nm). Sample GSH concentration was determined through a serial‐diluted GSH calibration curve (150 nM to 20 μM).

### ROS quantification

Oxidative stress in live cultures was assessed with CellROX Green (Life Technologies, #C10444), which remains non‐fluorescent until oxidized by intracellular ROS. The fluorescent signal intensity is proportional to the levels of intracellular free radicals. Cultures were plated in black optical bottom plates (Thermo Fisher, #NUN165305) and incubated in CellROX Green for 30 min after treatment, followed by 2× PBS washes prior to imaging. Images were captured on an Incucyte Zoom in phase contrast and green at 20× magnification (0.61 μm/pixel resolution) with three images per well. Mean Green Intensity was normalized to culture confluence for each image.

### Blinding and statistical analysis

Experimenter conducting the sample analysis for the GSH assay and western blot assays were blinded to the sample treatment details. Confluence, migration and caspase substrate experiments were automatically quantified through standardized algorithms and therefore not blinded. No randomization was performed to allocate samples in this study. There were no differences in sample size between the beginning of the experiments and their conclusion. This study was not pre‐registered.

Analyses were performed using Graphpad Prism (GraphPad Software Inc., San Diego, CA, USA). Treatment effects were assessed using one‐way or two‐way analysis of variance (anova) as relevant, followed by Bonferroni's multiple comparisons test where appropriate. All cell culture experiments were conducted with at least three independent biological replicates and at least two technical replicates each. Significance was accepted at *p* < 0.05 and data presented as mean ± standard error of mean (SEM) for biological replicates. Number of replicates (*n*) in figure legends indicates independent experiments, with averages of technical replicates within each experiment.

## Results

### Design and synthesis of Q‐PAC

**Figure 1:**
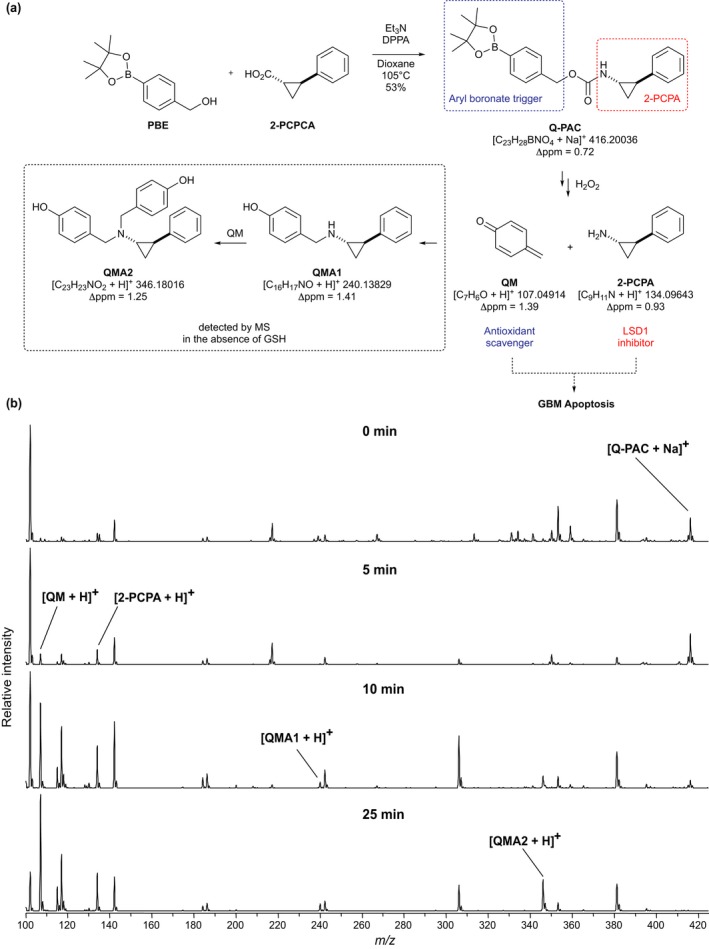
Quinone methide phenylaminocyclopropane (Q‐PAC) is activated by hydrogen peroxide (a) Activation of Q‐PAC by hydrogen peroxide liberates quinone methide (QM) and *trans*‐2‐phenylcyclopropylamine (2‐PCPA). In the absence of glutathione (GSH), subsequent formations of adducts QMA1 and QMA2 were detected by MS. (b) (+)ESI‐MS data were collected at selected times after Q‐PAC treatment with hydrogen peroxide. Sodiated Q‐PAC resulting from analysis of the untreated prodrug is labelled in the time‐zero spectrum at *m/z* 416. Peaks corresponding to activation products and adducts shown in panel (a) are also labelled. All other peaks have been accounted for as background with the exception of *m/z* 306. Accurate mass data (not shown) support assignment to the phenol derivative of Q‐PAC (sodium adduct), which is the structure obtained by boronate oxidation prior to breakdown to QM and 2‐PCPA.

The prodrug Q‐PAC was designed so that it would simultaneously release the LSD1 inhibitor 2‐PCPA and QM in the presence of high concentrations of H_2_O_2_. Q‐PAC was synthesized using a Curtius rearrangement as the key step and its chemical structure assigned by NMR and mass spectrometry (Fig. 1a and Figure S1 **)**. The presence of the carbamate linkage in Q‐PAC was confirmed by a ^13^C resonance at 156.8 ppm (Figure S1a) and the four methyl groups of the pinacol boronic ester were observed as a 12H singlet at 1.33 ppm in the ^1^H NMR (Figure S1b). This spectral data, in combination with a shift of the methylene protons from 4.72 ppm in the starting 4‐(hydroxymethyl)phenylboronic acid pinacol ester to 5.12 ppm in Q‐PAC provided confirmation that the coupling of the two active fragments of the prodrug had taken place. Using the same route we also prepared control compound QAC, which would generate quinone methide like Q‐PAC, but releases LSD1 inactive cyclopropylamine in place of 2‐PCPA. Similar to Q‐PAC, the formation of QAC was indicated by the OCH_2_ signal at 5.11 ppm in ^1^H NMR (Figure S2a) and carbamate signal at 157.0 ppm in ^13^C NMR (Figure S2b). In QAC the aminocyclopropane‐generating fragment lacks a phenyl group, which is essential for LSD1 inhibition, and QAC is therefore used as a control to demonstrate that the QM and LSD1 inhibitor components of Q‐PAC act synergistically.

### Q‐PAC is activated by hydrogen peroxide

It was envisaged that prodrug Q‐PAC activation with H_2_O_2_ would yield QM and 2‐PCPA (Fig. 1a). In the absence of GSH, QM can react with 2‐PCPA to yield adduct QMA1, which can then further react with another QM to form adduct QMA2 (Fig. 1a). We used positive‐mode electrospray ionization mass spectrometry (+ESI‐MS) to examine this activation process. Prior to H_2_O_2_ addition to a Q‐PAC solution a signal detected at *m/z* 416 is assigned to [Q‐PAC + Na]^+^ (Fig. 1b). Under identical instrument conditions the appearance of *m/z* 107 and 134 following 5 min treatment with H_2_O_2_ indicates the presence of initial products QM and 2‐PCPA, respectively (Fig. 1b). Longer reaction times (Fig. 1b, 10 min and 25 min) show further relative increase in QM and 2‐PCPA and production of QMA1 and QMA2 adducts evidenced by *m/z* 240 and 346 signals respectively. These results are consistent with the activation mechanism and adduct formation illustrated in Fig. 1(a). Experiments were repeated on a LTQ Orbitrap XL for high‐resolution mass analysis to further support assignment. Figure 1(a) includes the exact mass for each compound and the corresponding errors calculated using the accurate masses acquired from averaged Orbitrap analyses (data not shown); all errors fall within 2 ppm.

### The prodrug Q‐PAC reduces migration and viability of U87 glioblastoma cells

**Figure 2:**
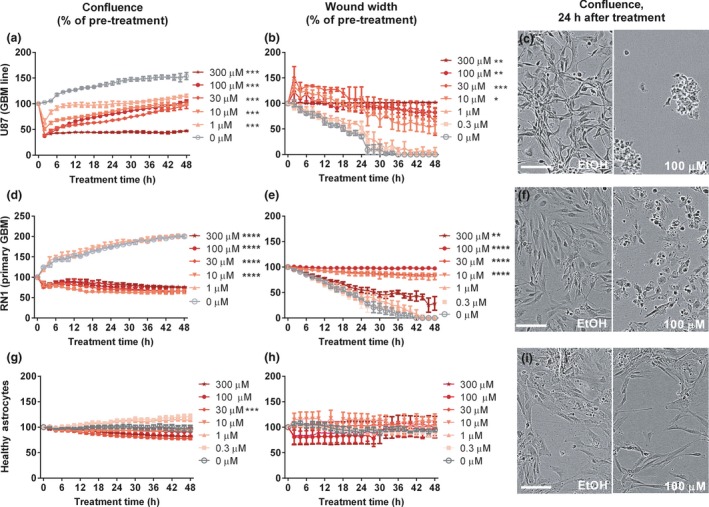
Quinone methide phenylaminocyclopropane (Q‐PAC) impairs mobility of primary glioblastoma (GBM) cells but not healthy astrocytes. Algorithm‐based confluence (*n* = 6) and 2D migration (*n* = 4) analysis of phase‐contrast microscope images of GBM cultures (a–f) and primary human astrocytes (g‐i) treated with Q‐PAC. Data represent mean ± SEM, *p < 0.05, **p < 0.01, ***p < 0.001, ****p < 0.0001 compared to vehicle control. (c, f, i) Representative images of U87 cultures treated with Q‐PAC. Cultures treated with vehicle (EtOH) or 100 μM Q‐PAC were captured in phase‐contrast images 24 h after treatment at 10× magnification (scale bar = 50 μm).

To investigate the two‐pronged approach of Q‐PAC against GBM, we first explored its anti‐cancer properties in the immortal U87 cell line, commonly used to assess novel GBM treatments. Q‐PAC treatment dose dependently reduced U87 confluence within 48 h (*F*(7,42) = 73.94, *p* < 0.0001; Fig. 2a). Q‐PAC treatment also reduced the migration (*F*(6,13) = 16.93, *p* < 0.0001; Fig. 2b) and invasion through matrigel (*F*(4,14) = 5.324, *p* = 0.0081, Figure S3e) of U87 cells, and reduced viability after 48 h treatment (*F*(6,28) = 9.47, *p* < 0.0001, Figure S3f). Cells responded within 4 h to Q‐PAC, showing a prominent change in morphology (Fig. 2c) and migration at concentrations above 1 μM.

To identify the contribution of the distinct components of Q‐PAC to the observed responses, we assessed the need for QM (using 2‐PCPA) and the requirement for LSD1 inhibition (using a phenyl‐free form of Q‐PAC (QAC) that would release QM and non‐LSD1 inhibitor cyclopropylamine (CPA)). Neither 2‐PCPA, CPA nor QAC affected the culture viability (Figure S4b), confluence (Figure S4c–e) or migration ability (Figure S4f–h) at concentrations up to 300 μM for up to 48 h. These data demonstrate that combining QM with 2‐PCPA provides the resulting compound with anti‐cancer properties, which neither of the individual components possesses.

### Q‐PAC shows higher selectivity for glioblastoma cells over healthy astrocytes

To probe for the selectivity of Q‐PAC against GBM cells, we evaluated the treatment response in primary GBM cultures and primary cerebral astrocyte cultures (human and mouse). The GBM cultures were grown from untreated biopsy samples of three different GBM subtypes (RN1: classical, JK2: proneural, SJH1: neural), which have been characterized previously (Day *et al*. 2013). Q‐PAC dose dependently reduced the culture confluence in the primary GBMs (*p* < 0.001 for each line, Fig. 2d and Figure S3a and c), and impaired migration in the scratch wound assay (*p* < 0.001 for each line, Fig. 2e and Figure S3b and d). While the three primary GBMs show different proliferation and migration rates, Q‐PAC treatment impaired both characteristics at concentrations above 10 μM. GBM culture viability dropped within 48 h of Q‐PAC treatment at concentrations of 30 μM and above (Figure S3f). Importantly, the potent LSD1 inhibitor Triazole 6 (Kutz *et al*. 2014) had no effect on viability, confluence or caspase activity in the primary GBM RN1 cells, and only reduced migration at 100 μM (Figure S5). In contrast to the GBM cultures, healthy astrocytes treated with Q‐PAC at concentrations up to 300 μM for 48 h showed no reduction in cell viability (*F*(6,53) = 0.56, *p* = 0.76; Figure S3f), or a change in their migratory behaviour (*F*(6,12) = 0.47, *p* = 0.82; Fig. 2h). Confluence of primary astrocyte cultures differed following Q‐PAC treatment (*F*(6,38) = 15.3, *p* < 0.0001; Fig. 2g), at 30 μM Q‐PAC (80.1% of vehicle after 48 h, *p* < 0.001; Fig. 2g), but to a lesser extent than for any of the GBM cultures at the same concentration (RN1: 32.7%, JK2: 64.6% and SJH1: 62.7% of vehicle). These results demonstrate the higher vulnerability of GBM cells to Q‐PAC treatment compared to healthy astrocytes *in vitro*.

### Q‐PAC causes apoptosis through GSH reduction following H3K4 methylation

Q‐PAC combines the LSD1 inhibitor 2‐PCPA with the glutathione scavenger QM. While GBM cultures reacted strongly to Q‐PAC treatment, neither 2‐PCPA nor QAC treatment resulted in such a response. We therefore investigated the mechanisms underlying the Q‐PAC treatment effect, whether it affects the epigenetic profile of histones or performs its functions through other mechanisms.

### Q‐PAC increases H3K4 mono and dimethylation without affecting H4 acetylation

**Figure 3:**
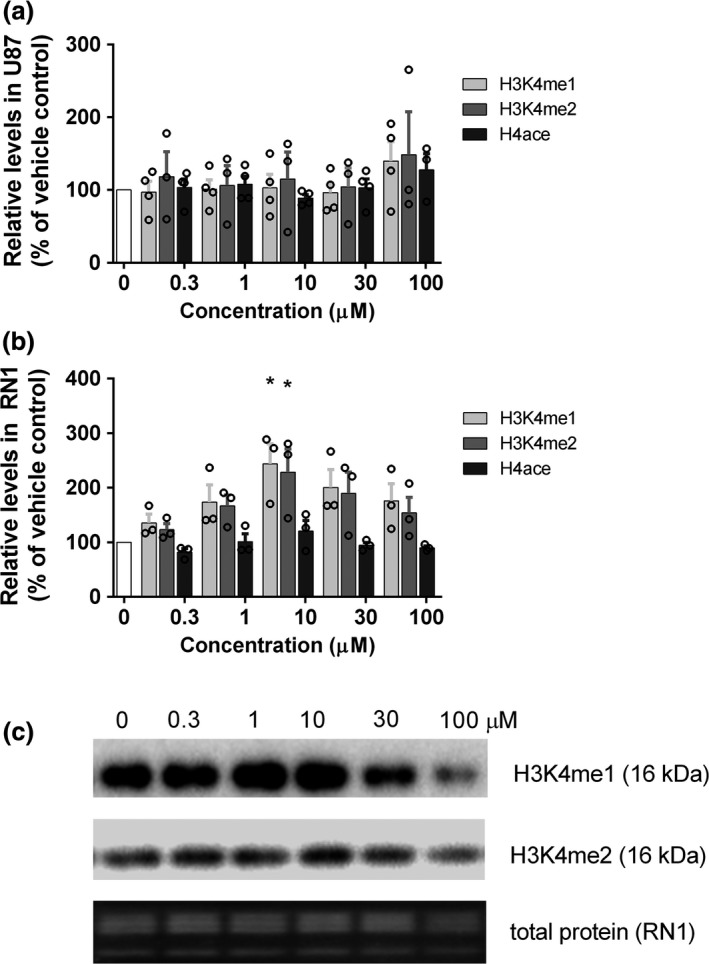
Quinone methide phenylaminocyclopropane (Q‐PAC) increases histone 3 lysine 4 (H3K4) dimethylation in primary glioblastoma cells. H3K4me1, H3K4me2 and H4ace levels quantified via immunoblotting in U87 (a, *n* = 4) and primary human glioblastoma cells (b, *n* = 3) after 4 h treatment with Q‐PAC (0–100 μM), adjusted to total loaded protein. (c) Representative immunoblot for RN1 cell samples, blotted for H3K4me1 (detected at 15 kDa), H3K4me2 (detected at 16 kDa) and total protein (segment depicting 8–20 kDa) for each Q‐PAC concentration (0–100 μM). Data represent mean ± SEM, *p < 0.05 compared to vehicle control.

Q‐PAC was designed to be selective for GBM over healthy cells via LSD1 inhibition as increased LSD1 protein levels have been observed in GBM (Singh *et al*. 2011; Sareddy *et al*. 2013; Zheng *et al*. 2015). We assessed the LSD1 inhibition properties of Q‐PAC, in comparison to 2‐PCPA and the potent LSD1 inhibitor Triazole 6, showing that Q‐PAC has a similar LSD1 inhibition profile as 2‐PCPA (Figure S6). We consequently quantified LSD1 levels in our cultures. In line with published results, the protein expression levels differed between culture types (*F*(5,12) = 17.24, *p* < 0.0001; Figure S7a and b), with RN1 and SJH1 cultures expressing higher levels of LSD1 compared to healthy astrocytes, while U87 and JK2 expression levels did not differ from healthy astrocytes. We quantified H3K4 mono (me1) and dimethylation (me2), as well as H4 pan acetylation, after treating U87 (low LSD1 levels) and primary RN1 (high LSD1 levels) cultures for 4 h, as changes in migration and confluence were observed within 4 h in response to Q‐PAC treatment (Fig. 2). In U87 cells Q‐PAC had no effect on H3K4me1 (*F*(5,18) = 1.024, *p* = 0.43; Fig. 3a), H3K4me2 (*F*(5,12) = 0.25, *p* = 0.93; Fig. 3a) or H4 acetylation (*F*(5,17) = 1.13, *p* = 0.38; Fig. 3a). In primary glioblastoma RN1 cells, however, 4 h of Q‐PAC treatment showed a concentration‐dependent effect on H3K4me1 (*F*(5,12) = 3.18, *p* = 0.05; Fig. 3b and c), H3K4me2 (*F*(5,12) = 2.74, *p* = 0.07; Fig. 3b and c) but not pan H4 acetylation (*F*(5,12) = 1.51, *p* = 0.26; Fig. 3b and c). Both me1 and me2 of H3K4 peaked at 10 μM Q‐PAC, while not differing to control levels at lower or higher concentrations (Fig. 3b). When assessing the selectivity of Q‐PAC for GBM, we found that 10 μM Q‐PAC after 4 h did not affect H3K4me2 (t = 0.26, df = 6, *p* = 0.814; data not shown) in healthy astrocytes (H3K4me1 was not detected in the healthy astrocytes).

### Q‐PAC triggers concentration‐dependent caspase 3/7 activity increase in GBM cells

LSD1 inhibitors have been shown to cause cell cycle arrest in breast cancer cells (Pollock *et al*. 2012). We therefore investigated whether cell cycle arrest is contributing to the reduction in cell viability and confluence observed in the GBM cultures after Q‐PAC treatment. After quantifying the expression of minichromosome maintenance 2 (MCM2), part of the DNA replication machinery that is only expressed in proliferating cells (Williams and Stoeber 2007), we found that 48 h of Q‐PAC treatment did not alter the proportion of MCM2‐positive U87 or primary GBM cells (Figure S7c). Thus, the reduction in viable cells appears not to be caused by driving cancer cells out of the cell cycle.

**Figure 4:**
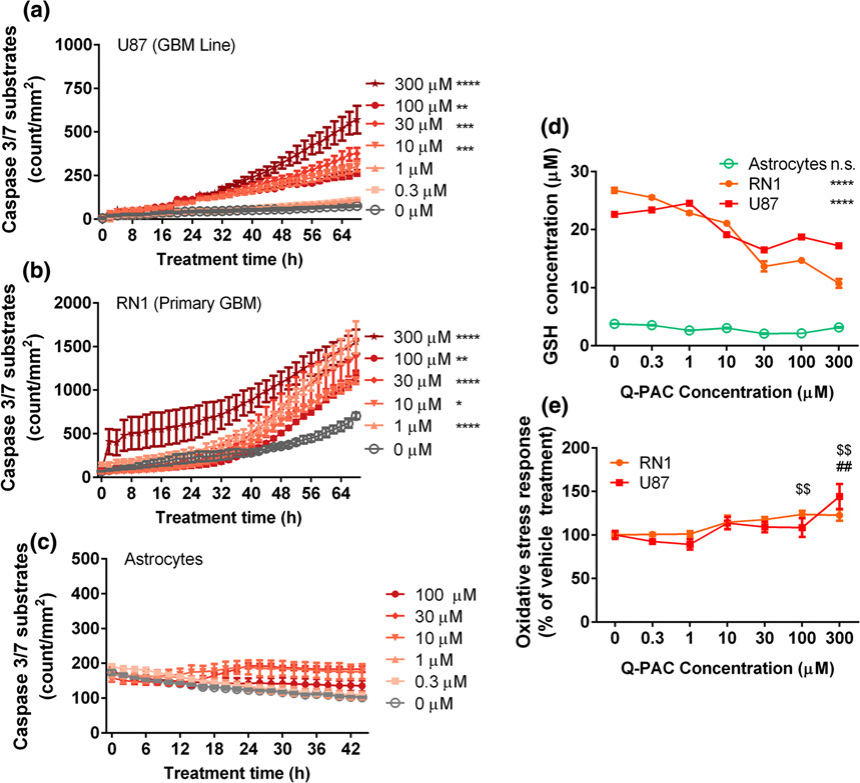
Quinone methide phenylaminocyclopropane (Q‐PAC) triggers apoptosis and oxidative stress in primary glioblastoma (GBM) cells but not healthy astrocytes. Apoptosis of U87 (a), primary human GBM (b) and primary human astrocyte cultures (c) treated with Q‐PAC. Apoptosis was quantified through counting of green‐fluorescent caspase 3/7 substrates per mm^2^ in microscope images at 20× magnification over time (*n* = 3 per concentration and culture). (d) Intracellular GSH concentration was quantified via fluorometric assay 4 h after treatment with Q‐PAC (0–300 μM) in U87, primary GBM cells (RN1) and primary astrocytes (*n* = 3 per concentration and cell type). (e) Oxidative stress levels were quantified via a cell‐permeant fluorogenic probe 4 h after treatment with Q‐PAC (0–300 μM) in U87 and primary GBM cells (RN1), normalized to vehicle‐treated cultures (*n* = 3 per concentration and cell type). Data represent mean ± SEM, *p < 0.05, **p < 0.01, ***p < 0.001, ****p < 0.0001 compared to vehicle control; $$*p* < 0.01 compared vehicle control (RN1); ##*p* < 0.01 compared to vehicle control (U87).

Apoptosis is a possible alternative explanation for the reduction in cell viability after Q‐PAC treatment, especially as increased apoptosis following the inhibition of LSD1 has been shown in colon and blood cancer cells (Wen *et al*. 2012; Ding *et al*. 2013). To assess whether Q‐PAC treatment results in apoptosis, we monitored caspase 3/7 activity in treated GBM cells and healthy astrocytes. Q‐PAC dose dependently increased caspase 3/7 activity in U87 (*F*(6,11) = 24.30, *p* < 0.0001; Fig. 4a), RN1 (*F*(5,22) = 6.2, *p* = 0.001; Fig. 4b), SJH1 (*F*(6,7) = 4.83, *p* = 0.028; Figure S7d) and JK2 (*F*(5,6) = 5.7, *p* = 0.027; Figure S7e) cultures, consistent with an increase in apoptosis in these cell types, without affecting caspase 3/7 activity in healthy astrocytes (*F*(5,12) = 2.67, *p* = 0.08; Fig. 4c).

### Q‐PAC reduces GSH levels in GBM cells

Work by Noh *et al*. (2015) shows that an increase in caspase 3/7 activity in cancer cells can be the result of GSH depletion through scavengers such as QM, a component of Q‐PAC (Fig. 1). We consequently quantified GSH and ROS levels following Q‐PAC treatment for 4 h, as this time point reflects the onset of treatment effects on confluence and migration (Fig. 2). GSH levels dose dependently decreased in U87 and RN1 cells, but not in healthy astrocytes (Fig. 4d), after Q‐PAC treatment. At the same time, Q‐PAC treatment increased ROS levels in U87 (*F*(7,33) = 5.03, *p* < 0.001; Fig. 4e) and RN1 cultures (*F*(6,28) = 5.76, *p* < 0.001; Fig. 4e) after 4 h in a concentration‐dependent manner.

## Discussion

Here, we describe the synthesis of the novel anti‐GBM drug Q‐PAC and its characterization *in vitro*. We provide mechanistic evidence that the prodrug Q‐PAC undergoes oxidation of its boronate functionality to release the active components QM and 2‐PCPA. Our *in vitro* study shows that Q‐PAC: (i) reduces proliferation, migration, invasion, GSH levels and viability of GBM cells; (ii) increases caspase 3/7‐mediated apoptosis, intracellular oxidative stress and H3K4 mono‐ and dimethylation; and (iii) shows a higher potency against GBM cells than healthy astrocytes. We provide the first *in vitro* evidence for a dual‐function strategy of possible LSD1 inhibition and GSH scavenging after activation by H_2_O_2_ as a selective treatment avenue against GBM.

The 2‐PCPA‐based compound Q‐PAC impaired several aspects of U87 cells at concentrations as low as 10 μM, while even 300 μM 2‐PCPA was ineffective against the immortal GBM cells, despite a similar potency against LSD1. 2‐PCPA treatment effects against breast cancer and neuroblastoma cells have been shown by several studies (Schulte *et al*. 2009; Lim *et al*. 2010), but required concentrations up to 20‐fold higher than the IC_50_ for 2‐PCPA (20.7 μM). Similarly, high concentrations of 2‐PCPA have been shown to be ineffective against other immortal GBM cultures, despite high LSD1 protein levels (Singh *et al*. 2011). Q‐PAC not only performed better than 2‐PCPA against GBM viability, but also reduced the migration and invasion phenotype of U87 cultures. Aggressive migration and invasion are key treatment challenges of GBM tumours (Demuth and Berens 2004). The suppression of cell migration and invasion following Q‐PAC application is thus a promising treatment characteristic of this novel compound. Critically, treatment with the two base components (2‐PCPA or QAC) was not effective against U87 cells, highlighting the need for the local activation of the dual‐action prodrug Q‐PAC.

Considering the marked difference in treatment response of GBM cells to Q‐PAC compared with 2‐PCPA, we questioned whether Q‐PAC had retained its LSD1 inhibitor function. While the enzymatic assay confirmed the inhibition of LSD1 by Q‐PAC, surprisingly, H3K4me1 and H3K4me2 (the primary targets of LSD1 (Lee *et al*. 2006)) appeared unaffected by Q‐PAC treatment in U87 cells. An inverted u‐shape concentration‐dependent effect on H3K4me1 and H3K4me2 was, however, observed in primary GBM cultures, with the peak difference at 10 μM. One reason for inhibiting LSD1 was to reduce the epigenetic suppression of tumour suppressor genes, as previously shown for LSD1 inhibitors in colorectal cancer (Huang *et al*. 2009). A reduction in cell proliferation would have been expected but did not occur in U87 or primary GBM cells following Q‐PAC treatment as suggested by a lack of an effect on MCM2 (Figure S5c). Together with the limited effect on global H3K4 methylation levels, this finding suggests that Q‐PAC causes only minor global methylation changes at these sites, but that H3K9me and gene‐specific methylation changes are likely (Schenk *et al*. 2012; Kidder *et al*. 2014; Zheng *et al*. 2015). In addition, LSD1 suppresses tumour suppressor protein 53 (p53) activity (Huang *et al*. 2007), with loss of LSD1 resulting in reduced colon cancer proliferation (Jin *et al*. 2013). Q‐PAC inhibition of LSD1 could thus result in reduced p53 promoter and protein methylation, enabling p53‐mediated apoptosis (Schuler *et al*. 2000; Scoumanne and Chen 2008), which could be assessed via chromatin immunoprecipitation sequencing in future experiments.

We designed Q‐PAC to be activated by H_2_O_2_, levels of which are higher in close proximity to GBM cells, and to separate into 2‐PCPA and QM. QM reportedly alkylates GSH, preventing its antioxidant function (Hagen *et al*. 2012; Marzenell *et al*. 2013). GBM cells up‐regulate GSH to compensate for their increased ROS production (Ogunrinu and Sontheimer 2010). Q‐PAC and other anti‐tumour drugs (including anti‐GBM compounds) therefore target this mechanism as a possible treatment avenue (Alexandre *et al*. 2006; Badr *et al*. 2013; Kohsaka *et al*. 2013; Noh *et al*. 2015). In our mass spectrometry assay, while not being a kinetic assay, we confirmed that prodrug Q‐PAC was able to undergo H_2_O_2_‐induced oxidative cleavage of its boronate functionality to QM, 2‐PCPA and QM‐derived adducts. This was critical given the desired dual action of the drug that required QM to reduce the antioxidant GSH while 2‐PCPA simultaneously inhibits LSD1. Our result is consistent with the report of Hagen *et al*. (2012), who demonstrated a similar breakdown of aminoferrocene‐based prodrugs with H_2_O_2_. Unlike Hagen *et al.,* however, we were able to directly detect QM, as well as its conjugate addition products. Given the concentration of GSH in cells is higher than the effective concentration of Q‐PAC (Khan *et al*. 2012), the desired reaction between GSH and QM would negate the formation of adducts QMA and therefore reduce the antioxidant ability of the cell. In support of this mechanism, we show that Q‐PAC concentration dependently reduces GSH levels in U87 and primary GBM cultures within 4 h of treatment, but not in primary astrocytes. Oxidative stress levels increased within the same treatment timeframe in U87 and primary GBM cells. Similar results have been reported from glioblastoma, prostate and colon cancer cell studies, where GSH scavenger treatment caused a reduction in GSH followed by an increase in ROS levels and apoptosis (Khan *et al*. 2012; Badr *et al*. 2013; Noh *et al*. 2015). Q‐PAC treatment likewise resulted in apoptosis, causing a concentration‐dependent increase in caspase 3/7 activity in U87 and primary GBM cultures without affecting caspase activity in primary astrocytes. Caspase 3/7 activity increased at Q‐PAC concentrations of 10 μM and above, while a 10‐fold higher concentration was required for the ROS level increase, indicating that GSH depletion can trigger apoptosis without measurable changes in ROS production (Khan *et al*. 2012). The lack of any treatment effects following the phenyl ring‐free Q‐PAC structure (QAC) treatment provides some support for the required combination of LSD1 inhibition and GSH quenching of this hybrid anti‐cancer drug. Furthermore, the lack of any 2‐PCPA effects on GBM cells, a potent monoamine amine oxidase inhibitor (Yang *et al*. 2007), suggests that monoamine oxidase inhibition is not the primary mode of action of Q‐PAC.

A marked feature of Q‐PAC across all culture assays has been the selectivity for GBM cells over healthy astrocytes. The limited effect against healthy astrocytes is likely because of three related differences in comparison to GBM cells: 1) Q‐PAC has been designed to be activated by high local H_2_O_2_ levels, which are found in the proximity of GBM cells, but not healthy cells (Reuter *et al*. 2010; Peng and Gandhi 2012); 2) the high metabolic activity of GBM cells results in increased ROS production and heavy reliance on the antioxidant properties of GSH (Ogunrinu and Sontheimer 2010; Noh *et al*. 2015), making GBM cells highly susceptible to GSH scavenging; and 3) LSD1 expression is higher in GBM cells than healthy cells, as shown in the present and previous studies, increasing the vulnerability to LSD1 inhibitor treatment (Schulte *et al*. 2009; Singh *et al*. 2011; Sareddy *et al*. 2013). While GBM cells would thus be affected by lower LSD1 inhibitor concentrations than healthy cells, irreversible LSD1 inhibitors would nonetheless run the risk of affecting LSD1 function in healthy cells, with cognitive deficits being reported after prolonged administration in mice (Neelamegam *et al*. 2012). Connecting the LSD1 inhibitor with the GSH scavenging mechanism through an aryl boronate H_2_O_2_ activation trigger appears to add a beneficial selectivity layer to Q‐PAC. LSD1 inhibitors for lung cancer have progressed to human trials, while pre‐clinical studies with cell and animal GBM models have been of mixed success (Singh *et al*. 2011; Sareddy *et al*. 2016), despite high LSD1 expression in the affected tissue. After observing the successful activation of Q‐PAC, we evaluated its ability to pass through the blood–brain barrier by calculating the log *BB* parameter (Clark 1999; Goodwin and Clark 2005). The calculated log BB value for Q‐PAC is 0.10, above the −0.3 cut‐off for blood–brain barrier permeability (Rodríguez‐Rodríguez *et al*. 2009), suggesting that Q‐PAC will pass through the blood–brain barrier. Our promising work thus needs to be followed up by assessing Q‐PAC in xenograft *in vivo* GBM mouse models for pharmacodynamics and pharmacokinetics as well as further assessing the potential role of 2‐PCPA.

In conclusion, we have designed and synthesized a novel prodrug that differs in concept from previous QM‐generating prodrugs in that it does not only rely on redox disruption in cancer cells. Rather, Q‐PAC targets the redox mechanism of the cancer cells, with a possible role for LSD1 inhibition that is subject to further investigation. We show that Q‐PAC reduces viability, migration and invasion, and triggers apoptosis selectively in glioblastoma cells but not healthy astrocytes. Thus, our double‐hit strategy exploiting LSD1 inhibition and GSH reduction in GBM forms the first fundamental step for a promising treatment approach.

## Author contributions

Conceptualization: C.H., L.O., M.E., Y.S.G.; Methodology: M.E., L.O., Y.S.G., C.H., A.M.; Validation: M.E., C.H., Y.S.G., A.M.; Formal Analysis: M.E., Y.S.G; Investigation: M.E., L.O., C.H., Y.S.G., A.M., D.C., A.H., G.S.; Resources: L.O., C.H., B.H., G.G., B.S.; Writing – Original Draft: M.E., L.O., C.H., Y.S.G.; Writing – Review & Editing: A.M., B.H., B.S.; Visualization: M.E., C.H., Y.S.G.; Supervision: L.O., C.H., M.E.; Project Administration: L.O., C.H.; Funding Acquisition: L.O., C.H.

## Supporting information


**Figure S1.** Spectral data confirming the coupling of the two active fragments of the prodrug Q‐PAC had taken place.
**Figure S2.** Spectral data confirming the coupling of the two active fragments of the prodrug QAC had taken place.
**Figure S3.** Q‐PAC impairs viability and mobility of primary GBM cells but not healthy astrocytes.
**Figure S4.** U87 cells show no response to the constituent parts of Q‐PAC.
**Figure S5.** The LSD1 inhibitor Triazole 6 has no effect on viability, confluence or caspase activity of the primary GBM RN1 cells.
**Figure S6.** Q‐PAC inhibits LSD1.
**Figure S7.** Prodrug Q‐PAC triggers apoptosis and oxidative stress in primary glioblastoma (GBM), without affecting proliferation regulation.

## Notes

^a^ The carbon directly attached to boron was not detected, likely as a result of quadropolar relaxation (Wray 1979).

^b^ Overlap of two signals.
