# Supplementary material for: Novel dual‐action prodrug triggers apoptosis in glioblastoma cells by releasing a glutathione quencher and lysine‐specific histone demethylase 1A inhibitor
Source: J Neurochem. 2019 Feb 3;149(4):535–50. doi: 10.1111/jnc.14655 (PMC6590141; doi:10.1111/jnc.14655)
Supplement: Supplementary file 1 — Figure S1. Spectral data confirming the coupling of the two active fragments of the prodrug Q‐PAC had taken place. Figure S2. Spectral data confirming the coupling of the two active fragments of the prodrug QAC had taken place. Figure S3. Q‐PAC impairs viability and mobility of primary GBM cells but not healthy astrocytes. Figure S4. U87 cells show no response to the constituent parts of Q‐PAC. Figure S5. The LSD1 inhibitor Triazole 6 has no effect on viability, confluence or caspase activity of the primary GBM RN1 cells. Figure S6. Q‐PAC inhibits LSD1. Figure S7. Prodrug Q‐PAC triggers apoptosis and oxidative stress in primary glioblastoma (GBM), without affecting proliferation regulation. [file JNC-149-535-s001.docx]

Supplementary Information

# Novel Dual-Action Prodrug Triggers Apoptosis in Glioblastoma Cells by Releasing a Glutathione Quencher and Lysine-specific Histone Demethylase 1A Inhibitor

Martin Engel, Yi Sing Gee, Dale Cross, Alan Maccarone, Benjamin Heng, Amy Hulme, Grady Smith, Gilles J. Guillemin, Brett W. Stringer, Christopher J. T. Hyland*, Lezanne Ooi*^,^

* Christopher Hyland, [chrhyl@uow.edu.au](mailto:chrhyl@uow.edu.au), Tel: + 61 24221 4953, Fax: +61 24221 4287
* Lezanne Ooi, [lezanne@uow.edu.au](mailto:lezanne@uow.edu.au), Tel: +61 24221 5865, Fax: +61 24221 8130

# Content

Supplementary Figures 2

# Supplementary Figures


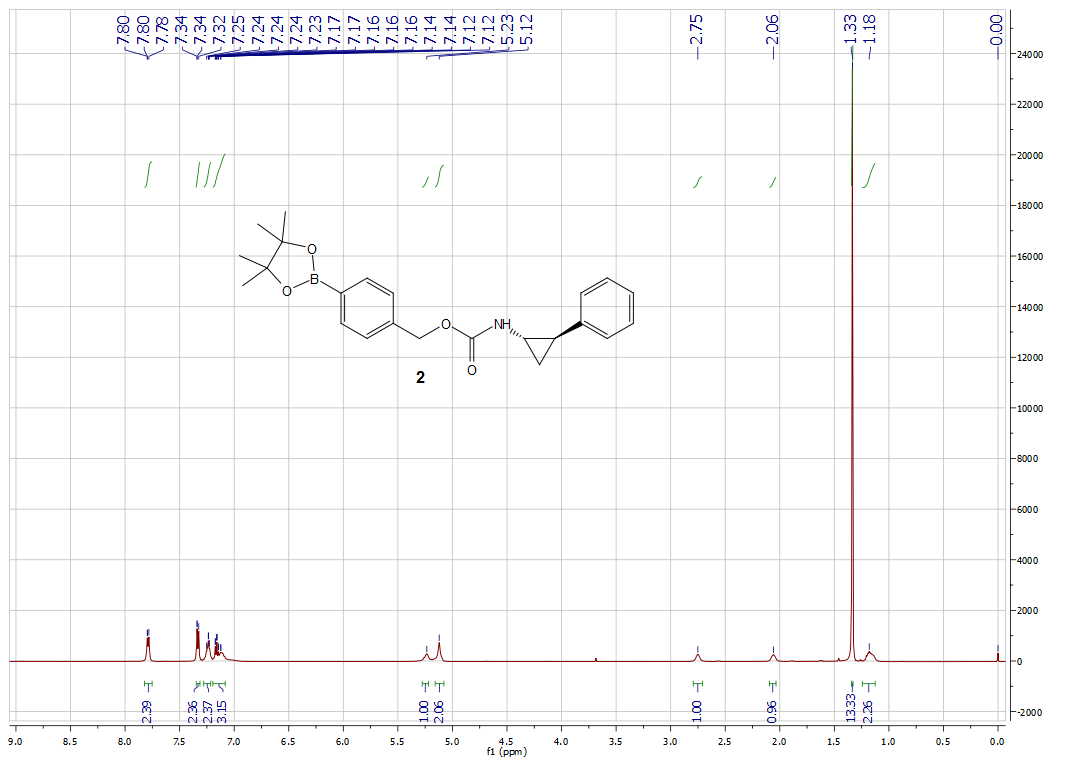


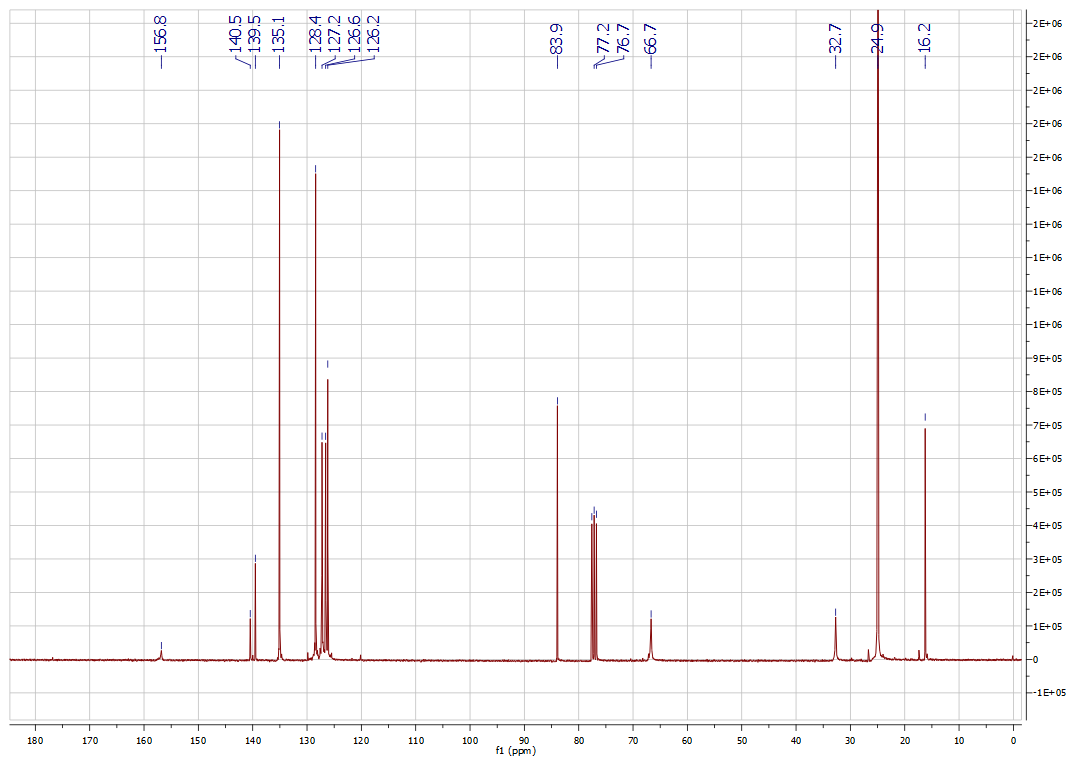


**Supplemental Figure 1: Spectral data confirming the coupling of the two active fragments of the prodrug Q-PAC had taken place.** (a) ^1^H NMR spectrum of Q-PAC recorded at 500 MHz in CDCl_3_. (b) ^13^C NMR spectrum of Q-PAC recorded at 75 MHz in CDCl_3_.


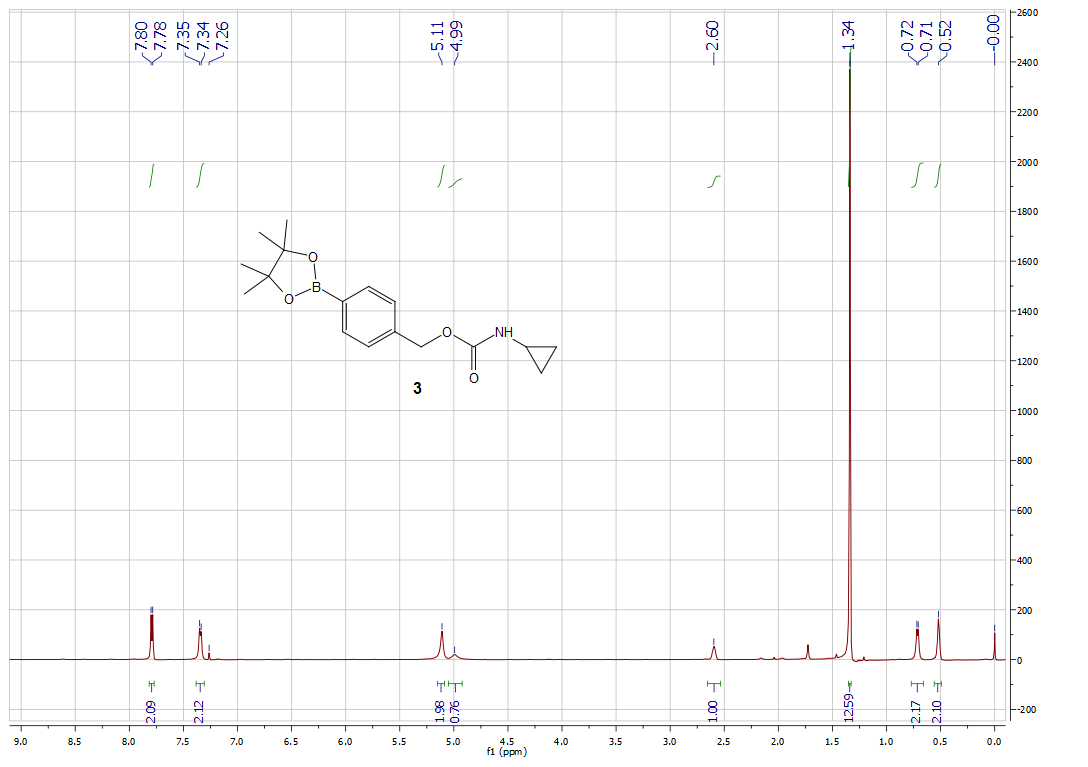


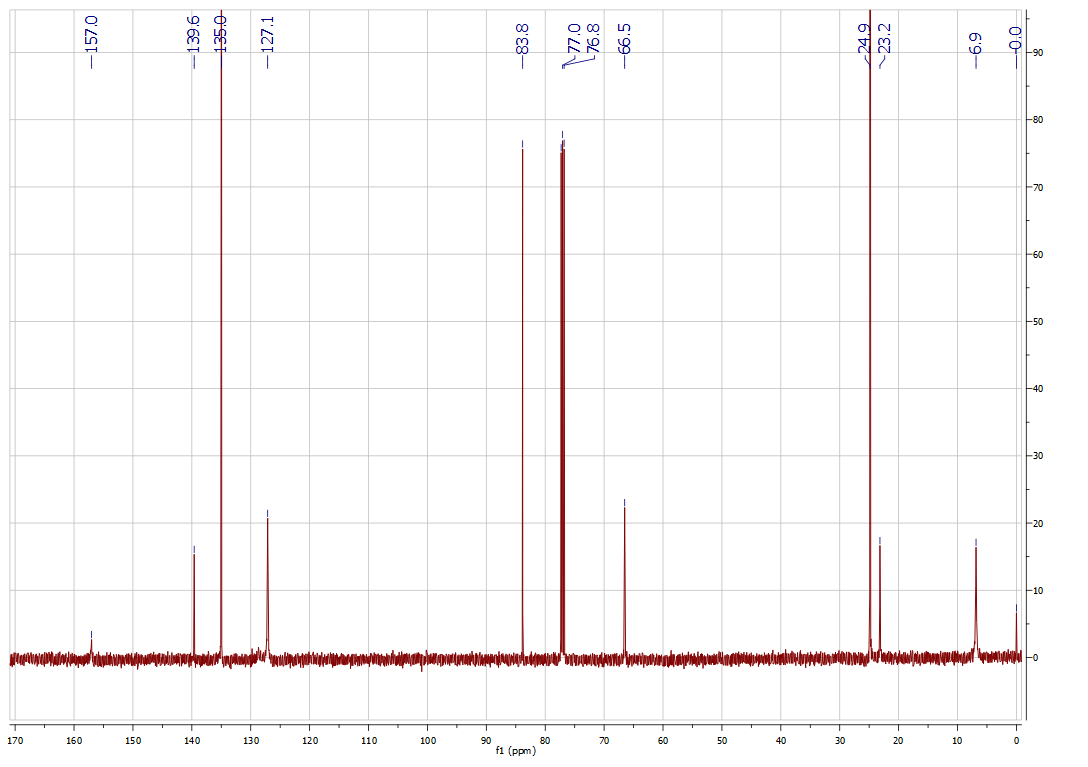


**Supplemental Figure 2: Spectral data confirming the coupling of the two active fragments of the prodrug QAC had taken place.** (a) ^1^H NMR spectrum of QAC recorded at 500 MHz in CDCl_3_. (b) ^13^C NMR spectrum of QAC recorded at 125 MHz in CDCl_3_.





**Supplemental Figure 3:** Q-PAC impairs viability and mobility of primary GBM cells but not healthy astrocytes. Confluence of primary human GBM (a, c) cultures treated with Q-PAC was quantified via an algorithm-based analysis of phase-contrast microscope images at 10x magnification over a 48 h period (n=3 per concentration & culture), normalised to culture confluence prior treatment. 2D migration of human GBM (b, d) cultures treated with Q-PAC. Scratch wound width was quantified through algorithm-based analysis of phase-contrast microscope images at 10x magnification following scratch wound and Q-PAC treatment (n=3 per concentration and culture), normalised to wound width prior treatment. (e) 3D invasion of U87 cells treated with Q-PAC. Invasion through matrigel was measured via electrical impedance every 30 min over a 24 h period following Q-PAC treatment (n=4 per concentration). (f) Viability of primary cultures treated with Q-PAC for 48 h. Cell viability was quantified via a Resazurin-based fluorometric assay in cultures treated for 48 h (n = 4). Readings were normalised to media-only cultures. Data represent mean ± SEM, * P<0.05, ** P<0.01, ***P<0.001, ****P<0.0001 compared to vehicle control.





**Supplemental Figure 4: U87 cells show no response to the constituent parts of Q-PAC.** (a) Chemical structure of 2- PCPA (2-phenylcyclopropylamine), QAC (quinone-aminocyclopropane), and CPA (cyclopropylamine) (b) U87 cultures were treated with the components of Q-PAC (2-PCPA, CPA) and the phenyl-free control compound QAC with their viability assessed after 24 h via a resazurin-based fluorometric assay. Confluence (c-e) was quantified via an algorithm-based analysis of phase-contrast microscope images at 10x magnification over a 48 h period, normalised to culture confluence prior to treatment. 2D migration of cultures treated with QAC (f), CPA (g) or 2-PCPA (h) was assessed via wound confluence as quantified through algorithm-based analysis of phase-contrast microscope images at 10x magnification following scratch wound and drug treatment. Data represent mean ± SEM, n=4 independent replicates per concentration.





**Supplemental Figure 5:** The LSD1 inhibitor Triazole 6 has no effect on viability, confluence or caspase activity of the primary GBM RN1 cells. (a) Viability of RN1 cultures treated with Q-PAC for 48 h. Cell viability was quantified via a resazurin-based fluorometric assay in cultures treated for 48 h (n = 3). Readings were normalised to media-only cultures. (b) Confluence of RN1 cultures treated with Q-PAC was quantified via algorithm-based analysis of phase-contrast microscope images at 10x magnification over a 48 h period (n=3 per concentration), normalised to culture confluence prior treatment. (c) Apoptosis of primary human GBM RN1 cultures treated with Triazole 6. Apoptosis was quantified through counting of green-fluorescent caspase 3/7 substrates per mm^2^ in microscope images at 20x magnification over time (n=3 per concentration & culture). 2D migration of human GBM (d) cultures treated with Q-PAC. Scratch wound width was quantified through algorithm-based analysis of phase-contrast microscope images at 10x magnification following scratch wound and Q-PAC treatment (n=3 per concentration), normalised to wound width prior treatment. Data represent mean ± SEM, * P<0.05 compared to vehicle control.





**Supplemental Figure 6:** Q-PAC inhibits LSD1. The LSD1 inhibition potential of Q-PAC, QAC (quinone-aminocyclopropane), 2-PCPA (2-phenylcyclopropylamine) and Triazole 6 was assessed via a fluorometric assay (n=2 reactions per concentration and drug). Non-linear regression curve fitted to the mean ± SD.





**Supplemental Figure 7:** Prodrug Q-PAC triggers apoptosis and oxidative stress in primary glioblastoma (GBM), without affecting proliferation regulation. (a) Lysine-specific histone demethylase 1A (LSD1) protein levels were quantified via immunoblotting (n=3 per cell type) in human astrocytes and GBM cells (established cell line: U87; primary patient samples: RN1, SHJ1, JK2) normalised to total protein content of each sample. ** P<0.01 *** P<0.001 compared to astrocytes. (b) Representative immunoblot for LSD1 (detected at 110 kDa) and total protein for each cell type. (c) Cells positive for MCM2, part of the DNA replication machinery that is only expressed in proliferating cells, were counted in cultures treated with Q-PAC (0 to 300 µM) for 48 h (n=4 per concentration and type). MCM2-positive cells were quantified via antibody-based immunofluorescence labelling in microscope images at 20x magnification and presented as % of total cell number. (d, e) Apoptosis of primary human GBM cultures treated with Q-PAC. Apoptosis was quantified through counting of green-fluorescent caspase 3/7 substrates per mm^2^ in microscope images at 20x magnification over time (n=3 per concentration & culture). Data represent mean ± SEM, * P<0.05, ** P<0.01, ***P<0.001, ****P<0.0001 compared to vehicle control.
